# Genetic differentiation and hybrid identification using microsatellite markers in closely related wild species

**DOI:** 10.1093/aobpla/plv084

**Published:** 2015-07-17

**Authors:** Caroline Turchetto, Ana Lúcia A. Segatto, Júlia Beduschi, Sandro L. Bonatto, Loreta B. Freitas

**Affiliations:** 1 Laboratory of Molecular Evolution, Department of Genetics, Universidade Federal do Rio Grande do Sul, PO Box 15053, Porto Alegre, Brazil; 2 Laboratory of Genomic and Molecular Biology, Pontifícia Universidade Católica do Rio Grande do Sul, Ipiranga 6681, 90610-001 Porto Alegre, RS, Brazil

**Keywords:** EST-SSR markers, genetic differentiation, hybridization, *Petunia axillaris*, *Petunia exserta*, wild genetic diversity

## Abstract

Identifying the genetic basis of speciation is critical for understanding the evolutionary history of closely related wild species. Recently diverged species facilitate the study of speciation because many genetic and morphological characteristics are still shared by the organisms under study. The *Petunia* genus grows in South American grasslands and comprises both recently diverged wild species and commercial species. In this work, we analysed two closely related species: *Petunia exserta*, which has a narrow endemic range and grows exclusively in rocky shelters, and *Petunia axillaris*, which is widely distributed and comprises three allopatric subspecies. *Petunia axillaris* ssp. *axillaris* and *P. exserta* occur in sympatry, and putative hybrids between them have been identified. Here, we analysed 14 expressed sequence tag-simple sequence repeats (EST-SSRs) in 126 wild individuals and 13 putative morphological hybrids with the goals of identifying differentially encoded alleles to characterize their natural genetic diversity, establishing a genetic profile for each taxon and to verify the presence of hybridization signal. Overall, 143 alleles were identified and all taxa contained private alleles. Four major groups were identified in clustering analyses, which indicated that there are genetic distinctions among the groups. The markers evaluated here will be useful in evolutionary studies involving these species and may help categorize individuals by species, thus enabling the identification of hybrids between both their putative taxa. The individuals with intermediate morphology presented private alleles of their both putative parental species, although they showed a level of genetic mixing that was comparable with some of the individuals with typical *P. exserta* morphology. The EST-SSR markers scattered throughout the *Petunia* genome are very efficient tools for characterizing the genetic diversity in wild taxa of this genus and aid in identifying interspecific hybrids based on the presence of private alleles. These properties indicate that these markers will be helpful tools in evolutionary studies.

## Abstract (precis)

Despite extensive morphological diversity and different floral syndromes, two wild *Petunia* species are closely related, present high genetic similarity, and field observations suggest natural hybridization between them. Here we described helpful tools for evolutionary studies addressing genetic population, interspecific hybridization, and plant speciation. Based on previously described microsatellites of *Petunia hybrida* we were able to identify private alleles characterizing species and their putative hybrids. These profiles could also be useful to study gene flow, population structure, genetic conservation and landscape.

## Introduction

Genetic diversity and population structures are relevant for understanding the evolutionary history, breeding systems, geographical distributions and ecological requirements of species. In evolutionary studies, molecular markers that differentiate close species and their putative hybrids greatly increase our understanding of the genetic basis of speciation and the effects of introgression on species integrity.

The *Petunia* genus encompasses 14 taxonomically accepted wild species, which are distributed exclusively in southern South America (Stehmann *et al*. 2009) and present low genetic variability (Ando *et al*. 2005; Kulcheski *et al*. 2006; Lorenz-Lemke *et al*. 2010; Fregonezi *et al*. 2013), and one artificial hybrid known worldwide, the commercial *Petunia hybrida* that is the result of crossings between *Petunia axillaris* and *Petunia interior* (Segatto *et al*. 2014*b*). Moreover, species of this genus are commonly used as a scientific model species because of their intrinsic features (Gerats and Vandenbussche 2005).

**Figure 1.**
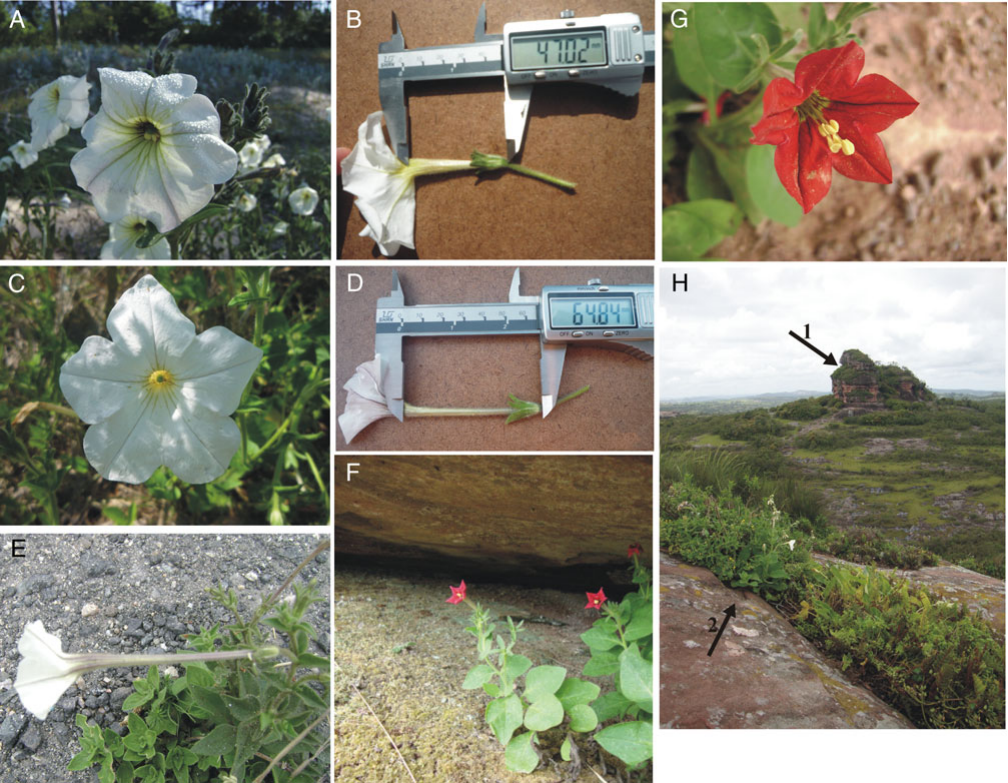
Morphologies and habitats of the taxa studied. (A and B) Typical *Petunia axillaris* ssp. *axillaris* morphology; (B) typical tube length is lower than other subspecies; (C and D) typical *Petunia axillaris* ssp. *parodii* morphology; (D) typical tube length; (E) typical *Petunia axillaris* ssp. *subandina* morphology; (F and G) typical *Petunia exserta* morphology and habitat (shelters); (H) landscape of Serra do Sudeste, highlighting the sandstone towers and habitats of *P. exserta* (black arrow 1) and *P. axillaris* ssp. *axillaris* (black arrow 2).

*Petunia axillaris* is the only species of the genus that presents white corollas (Stehmann *et al*. 2009) and is pollinated by hawkmoths (Ando *et al*. 1995; Venail *et al*. 2010; Klahre *et al*. 2011) (Fig. 1). This species is widespread in the Pampas grasslands of southern South America and currently consists of three taxonomically accepted subspecies: *P. axillaris* ssp. *axillaris*, *P. axillaris* ssp. *parodii* and *P. axillaris* ssp. *subandina*. These subspecies are morphologically distinguishable from each other by floral traits such as corolla tube length, relative positioning of the stamen and corolla, and corolla diameter (Ando 1996; Turchetto *et al*. 2014*a*, *b*) (Fig. 1). Plastid data analysis revealed low genetic differentiation among these subspecies, whereas the nuclear genome contained greater genetic variability (Turchetto *et al*. 2014*a*, *b*). The three subspecies occur in adjacent geographic regions, and the *P. axillaris* ssp. *axillaris* could be found in the same narrow region as another congeneric species, *Petunia exserta*, that presents red and bird-pollinated flowers (Lorenz-Lemke *et al*. 2006; Stehmann *et al*. 2009; Segatto *et al*. 2014*a*) (Fig. 1). Although they grow in sympatry, *P. exserta* and *P. axillaris* ssp. *axillaris* are partially isolated in adjacent microhabitats: whereas *P. exserta* plants grow only inside the shaded cracks (shelters) of sandstone towers where they are protected from direct rain and sunlight, *P. axillaris* ssp*. axillaris* individuals grow in open and sunny habitats (Fig. 1).

Previous studies addressing the genetic diversity of these species showed lower indices of genetic variability in *P. exserta*, in agreement with its narrow endemic status (Segatto *et al*. 2014*a*), and extensive divergence between the species. Moreover, natural hybridization between these species has been suggested based on their morphology as well as plastid and nuclear genomes data (Lorenz-Lemke *et al*. 2006; Segatto *et al*. 2014*a*).

Although the genetic diversity and population structure of these taxa have been previously addressed (Lorenz-Lemke *et al*. 2006; Segatto *et al*. 2014*a*; Turchetto *et al*. 2014*a*, *b*), here we provide a new approach and obtain more precise taxa characterization by using microsatellites derived from expressed sequence tag-simple sequence repeat (EST-SSR) markers.

Bossolini *et al*. (2011) developed, mapped and used 83 EST-SSRs to construct linkage maps in *Petunia* and these markers cover the seven chromosomes of *Petunia*. However, despite their great potential for evolutionary studies, the markers have not been used to access the genetic variability in wild *Petunia* species. Expressed sequence tag-simple sequence repeat markers offer advantages over genomic DNA-based markers: they can be applied to analyse the functional diversity; are more conserved among species; and are obtained from gene-rich regions of genome (Zhang *et al*. 2005; Guo *et al*. 2006). They can also be used efficiently to assess genetic relationships (Gupta and Rustgi 2004) and to compare closely related taxa (Fluch *et al*. 2011).

The main goals of this work were to evaluate genetic polymorphism in a subset of the *Petunia* EST-SSRs in wild *P. axillaris* subspecies and *P. exserta*, thereby identifying alleles private to each taxon and to employ these genetic profiles to detect putative natural hybrids.

## Methods

### Plant material and genomic DNA isolation

We collected young leaves of individuals from the three natural occurring *P. axillaris* subspecies and *P. exserta.* The young leaves were dried in silica gel and pulverized in liquid nitrogen to allow genomic DNA extraction with cetyltrimethylammonium bromide (CTAB) (Roy *et al*. 1992). Additionally, we included genomic DNA from the previously analysed natural hybrids (*P. axillaris* ssp. *axillaris* × *P. exserta*; Lorenz-Lemke *et al*. 2006) in our analyses (Table 1). The quality and quantity of the isolated DNA was estimated by measuring the absorbance at 260 and 280 nm on a Nanodrop Spectrophotometer (NanoDrop 1000 spectrometer, Thermo Scientific Corp., San Jose, CA, USA). The DNA was finally electrophoresed on a 1.0 % agarose gel using GelRed™ (Biotium, Inc., Hayward, CA, USA). Samples were stored until further study at −20 °C.

**Table 1.** Origin of the *Petunia* individuals analysed in this study. ICN, Herbarium of Universidade Federal do Rio Grande do Sul, Brazil; BHCB, Herbarium of Universidade Federal de Minas Gerais, Brazil; NA, not available; Solís-Neffa, Viviana Solis-Neffa, Universidad del Nordeste, Corrientes, Argentina; Kovalsky&Elías.

Taxa	Localities	Geographic coordinates	Voucher/collector	*n*
*P. axillaris* ssp. *axillaris*	1. Durazno/Uruguay	33°00′03″S/56°37′35″W	ICN164602	2
	2. San José/Uruguay	34°45′52″S/56°24′25″W	ICN158363	2
	3. Rocha/Uruguay	34°03′17″S/53°53′26″W	Solís-Neffa 2177	4
	4. Hulha Negra/Brazil	31°23′36″S/53°49′17″W	BHCB117009	2
	5. DonPedrito/Brazil	31°05′37″S/54°27′28″W	BHCB140474	2
	6. Caçapava do Sul/Brazil	30°50′21″S/53°31′18″W	BHCB140443	2
	7. Bagé/Brazil	30°58′37″S/53°36′18″W	BHCB140438	3
	8. Maldonado/Uruguay	34°54′48″S/55°02′45″W	ICN164604	2
	9. Caçapava do Sul/Brazil	30°50′24″S/53°30′01″W	BHCB75106	3
	10. Caçapava do Sul/Brazil	30°53′48″S/53°25′16″W	BHCB70028	9
	11. Caçapava do Sul/Brazil	30°50′02″S 53°29′59″W	NA	4
*P. axillaris* ssp. *parodii*	12. Tacuarembó/Uruguay	32°39′51″S/56°28′52″W	ICN164598	2
	13. Tacuarembó/Uruguay	31°48′42″S/56°12′59″W	ICN164599	2
	14. Alegrete/Brazil	29°56′13″S/56°04′20″W	BHCB102107	2
	15. Alegrete/Brazil	30°00′50″S/56°13′07″W	NA	2
	16. Corrientes/Argentina	30°13′00″S/59°23′39″W	Solís-Neffa 2197	2
	17. Formosa/Argentina	25°07′24″S/59°58′05″W	BHCB140477	2
	18. Salto/Uruguay	31°27′21″S/57°54′18″W	ICN158365	10
	19. Salto/Uruguay	31°20′03″S/57°19′33″W	ICN158366	7
	20. Artigas/Uruguay	30°34′08″S/56°36′16″W	ICN158373	10
*P. axillaris* ssp. *subandina*	21. Córdoba/Argentina	30°51′57″S/64°29′30″W	ICN164577	2
	22. Córdoba/Argentina	30°51′21″S/64°31′46″W	ICN164575	6
	23. Córdoba/Argentina	31°18′46″S/65°05′40″W	Kovalsky&Elías 5	1
	24. Córdoba/Argentina	31°45′02″S/64°55′50″W	BHCB140429	9
	25. Córdoba/Argentina	31°47′49″S/65°00′23″W	ICN164576	5
*P. exserta*	26. Caçapava do Sul/Brazil	30°50′18″S/53°30′38″W	NA	2
	27. Caçapava do Sul/Brazil	30°50′26″S/53°30′19″W	ICN158645	4
	28. Caçapava do Sul/Brazil	30°50′18″S/53°29′43″W	BHCB75107	4
	29. Caçapava do Sul/Brazil	30°49′52″S/53°30′10″W	BHCB79901	2
	30. Caçapava do Sul/Brazil	31°13′30″S/53°29′51″W	BHCB140448	2
	31. Caçapava do Sul/Brazil	31°13′39″S/53°30′30″W	ICN158537	2
	32. Caçapava do Sul/Brazil	30°50′20″S/53°31′17″W	BHCB140441	2
	33. Caçapava do Sul/Brazil	30°53′48″S/53°25′16″W	BHCB76030	5
	34. Caçapava do Sul/Brazil	30°50′18″S/53°30′38″W	NA	6
Total				126
Putative hybrids	35. Caçapava do Sul/Brazil	30°49′53″S/53°30′10″W	BHCB79902	2
	36. Caçapava do Sul/Brazil	30°53′48″S/53°25′16″W	BHCB79894	11
Total				13

### Expressed sequence tag-simple sequence repeat amplification

The genomic DNA of each individual was used to amplify the previously characterized EST-SSR markers (Bossolini *et al*. 2011; available online at http://www.botany.unibe.ch/deve/caps/index.html) scattered throughout the genome. Initially, we selected 34 primers based on their chromosomal location and polymorphic index content to test the cross-transferability among taxa and putative hybrids. Only 14 were successfully amplified and were subsequently used to estimate genetic variability. Polymerase chain reactions (PCRs) were conducted in a ﬁnal volume of 10 μL containing ∼10 ng of genomic DNA as a template, 200 μM of each dNTP (Invitrogen, Carlsbad, CA, USA), 1.7 pmol of ﬂuorescently labelled M13(-21) primer, 3.5 pmol of reverse primer, 0.35 pmol of forward primer with a 5′-M13(-21) tail, 2.0 mM MgCl_2_ (Invitrogen), 0.5 U of Platinum Taq DNA polymerase (Invitrogen) and 1× Platinum Taq reaction buffer (Invitrogen). The PCR conditions were as follows: an initial denaturation at 96 °C for 3 min; 32 cycles of 96 °C for 15 s, 50–52 °C for 30 s and 72 °C for 1 min; and a ﬁnal extension cycle at 72 °C for 7 min. The forward primers were FAM-, NED-, or HEX-labelled. The amplified product was visualized on a 2 % ultra-resolution agarose gel stained with 2 μL 0.001 % of GelRed (Biotium Inc.). The DNA fragments were denatured and size-fractionated using capillary electrophoresis on a MegaBACE 1000 automated sequencer (GE Healthcare Biosciences, Pittsburgh, PA, USA) with an ET-ROX 550 internal size ladder (GE Healthcare). The manufacturer's software was used to identify the alleles. The primer sequences, repeat motif, fragment size range as estimated by the number of base pairs, and respective annealing temperatures are shown in **Table S1 [Supporting Information]**.

### Statistical analysis

We used the FSTAT 2.9.3.2 software (Goudet 1995) to evaluate summary statistics such as the number of alleles per locus (*A*), gene diversity, allelic richness (RA) and number of private alleles (*E*) per locus. We estimated the frequencies of null alleles and the polymorphic information content using the CERVUS 3.0.3 software (Marshall *et al*. 1998; Kalinowski *et al*. 2007).

The tests for outlier loci were performed with the BAYESCAN 2.1 software (Foll and Gaggiotti 2008) based on the multinomial-Dirichlet model. The BAYESCAN software incorporates the uncertainty of allele frequencies due to small sample sizes. Selection was introduced by decomposing the locus-population *F*_ST_ coefficients into a population-specific component (β) shared by all loci and a locus-specific component (α) shared by all populations using a logistic regression approach. Selection is assumed to occur when the α component is necessary to explain the diversity observed at a locus.

To investigate the genetic similarity between taxa, we carried out a principal coordinates analysis (PCoA) using the GENALEX 6.4 software (Peakall and Smouse 2006, 2012). We used a distance matrix based on the alleles that were shared among individuals to depict the relationships among individuals in all taxa; the original data were bootstrapped 1000 times using the MICROSAT software (Minch *et al*. 1997). In addition, an unweighted neighbour-joining (NJ) tree (Saitou and Nei 1987) was constructed based on the mean bootstrapped matrix of microsatellite alleles shared among the 126 individuals calculated from 14 EST-SSRs using the MEGA6 software (Tamura *et al*. 2013).

### Evaluating hybrid individuals

We amplified and analysed 13 putative hybrid individuals from a contact zone between *P. axillaris* ssp. *axillaris* and *P. exserta* that presented intermediate morphological characters, such as corolla colour, reproductive organs size and relative position, as previously described  (Table 1) (Lorenz-Lemke *et al*. 2006). We conducted a discriminant analysis of principal components (Jombart *et al*. 2010) using the R package ADEGENET (Jombart 2008; R Development Core Team 2011) among the three *P. axillaris* subspecies, *P. exserta*, and the putative hybrids to identify and describe clusters of genetically related individuals. Discriminant analysis of principal components is better suited than STRUCTURE analysis (Pritchard *et al*. 2000) to unravel the underlying genetic structure in complex groups that are not necessarily populations (Jombart *et al*. 2010). Discriminant analysis of principal components is not based on pre-defined population genetics models and makes no assumptions about Hardy–Weinberg equilibrium or linkage disequilibrium. We set an *a priori* group number of five in the DAPC analysis. We used a factorial correspondence analysis (FCA) as performed in GENETIX version 4.05 (Belkhir *et al*. 2004) to plot multilocus genotypes and visualize species discreteness based on the most distinctive loci. In this analysis, each row (individuals) and each column (alleles) were depicted as a point. The hyperspace had as many dimensions as there were alleles for each locus, and the algorithm attempted to find independent directions in this hyperspace. We also ran a clustering analysis, as implemented in the STRUCTURE 2.3 software (Pritchard *et al*. 2000), to compare with the DAPC results. The parameters were correlated allele frequencies (Falush *et al*. 2003) and no prior population information was used. The number of groups (*K*) was evaluated from 1 to 10, with 10 independent runs per *K* value, to determine the maximum value of the posterior likelihood [ln *P*(*D*)] and the best value of *K*. Each run was performed using 2.5 × 10^5^ burn-in periods and 10^6^ Markov chain Monte Carlo repetitions after burn-in, and the convergence was assessed. The optimal value of *K* was calculated using the maximum value of Δ*K* (Evanno *et al*. 2005). We used the CLUMPP 1.1.2 software to summarize the results of the optimal *K* value based on the average pairwise similarity of individual assignments across runs using Greedy's method and the *G*′ statistic (Jakobsson and Rosenberg 2007). We used the DISTRUCT 1.1 software (Rosenberg 2004) to visualize the STRUCTURE results after processing with the CLUMPP software. Substructures within each main cluster were detected by the same approach using STRUCTURE.

## Results

### Primer validation and cross-transferability

Fourteen of the 34 previously described loci reached our criteria for markers that had the potential to discriminate among the four wild *Petunia* taxa and their putative interspecific hybrids.

The 14 EST-SSR loci revealed 143 alleles in 126 sampled individuals (Table 2). *Petunia axillaris* ssp. *axillaris* presented the highest diversity index, whereas *P. axillaris* ssp*. subandina* was the least diverse (Tables 2 and 3). The combined probability of identity for the 14 EST-SSR loci was almost zero for all taxa, which indicated that two unrelated individuals would not share the same multilocus genotype. The PIC values (Table 3) ranged from 0.42 (PM184) to 0.93 (PM177), which showed that these markers are polymorphic across taxa. We found private alleles in all taxa, with a higher value for *P. axillaris* ssp*. axillaris* (22 alleles), followed by *P. exserta* and *P. axillaris* ssp. *parodii* (nine alleles each) and *P. axillaris* ssp. *subandina* (seven alleles). The majority of the private alleles found in *P. exserta* or *P. axillaris* ssp*. axillaris* was present in their putative hybrids. In total, 12 private alleles were shared between *P. axillaris* ssp. *axillaris* (eight alleles) or *P. exserta* (four alleles) and their hybrids **[see Supporting Information—Table S2]**. Moreover, hybrid individuals shared one private allele found in *P. axillaris* ssp. *subandina,* which grows far ∼1000 km. The PM21 and PM88 loci presented two and one private alleles in the putative hybrids, respectively, which suggests that these alleles came from parental plants that were not sampled in our study **[see Supporting Information—Table S2]**.

**Table 2.** Genetic diversity of 126 *Petunia* individuals as revealed by 14 EST-SSR loci.

Taxon	Sample size	Alleles/species	Alleles/locus	Gene diversity/locus
*P. axillaris* ssp. *axillaris*	35	108	7.7	0.72
*P. axillaris* ssp. *parodii*	39	83	5.9	0.61
*P. axillaris* ssp. *subandina*	23	59	4.2	0.60
*P. exserta*	29	73	5.4	0.60
Total (all datasets)	126	143	10.2

**Table 3.** The summary statistics of the 14 EST-SSR loci estimated for each locus and each taxon. SR, size range of alleles; PIC, polymorphic information content; A, number of alleles; RA, allele richness; E, number of private alleles. The number of private alleles shared between taxa and hybrids individuals was presented in parenthesis.

Locus	SR	PIC	*P. axillaris* ssp. *axillaris*			*P. axillaris* ssp. *parodii*			*P. axillaris* ssp. *subandina*			*P. exserta*		
			A	RA	E	A	RA	E	A	RA	E	A	RA	E
PM101	240–276	0.64	8	6.88	3	4	3.81	0	3	3.00	0	3	2.89	0
PM188	115–151	0.82	8	7.63	3(2)	7	6.80	1	4	4.00	0	6	5.99	1(1)
PM195	190–229	0.67	5	4.94	1	5	4.12	1	3	3.00	0	3	3.00	0
PM21	125–137	0.53	5	4.85	2(1)	3	2.81	0	3	3.00	0	2	2.00	0
PM88	142–174	0.60	5	4.49	1	6	5.96	1	2	2.00	0	6	5.96	1(1)
PM183	124–180	0.84	14	12.43	4(1)	12	9.64	3	5	5.00	0	9	8.48	2
PM191	164–176	0.47	4	3.95	1	4	3.92	0	2	2.00	0	2	2.00	0
PM8	163–191	0.76	8	7.18	3(2)	4	4.00	0	2	2.00	0	2	2.00	0
PM173	157–196	0.77	12	10.05	4(1)	4	3.96	0	5	5.00	1	7	6.93	0
PM74	186–200	0.55	4	4.00	0	4	3.74	1	2	2.00	0	2	2.00	0
PM167	279–312	0.83	11	10.32	0	8	7.58	0	3	3.00	0	7	6.89	1(1)
PM177	202–260	0.93	12	11.07	0	13	11.19	1	14	13.87	5(1)	14	12.92	3(1)
PM192	224–260	0.80	8	7.81	0	7	6.69	1	7	6.96	0	9	8.73	1
PM184	90–102	0.42	4	3.58	0	2	1.99	0	4	4.00	1	4	3.93	0
Mean		0.69	8	7.01	1.57	6	5.44	0.64	4	4.20	0.43	5	5.26	0.64

The frequency of null alleles varied from 20 to 79 % across all taxa. Null alleles were present at high frequency (>5 %) in all taxa. The tests for outlier loci among all 126 individuals revealed that PM177 (*P* = 1), PM88 (*P* = 0.829), PM74 (*P* = 0.983) and PM192 (*P* = 1) were under selection with negative α values, which suggests the occurrence of a balancing or purifying selection. However, this result should be considered carefully because we did not sample a representative population.

### Genetic similarity

**Figure 2.**
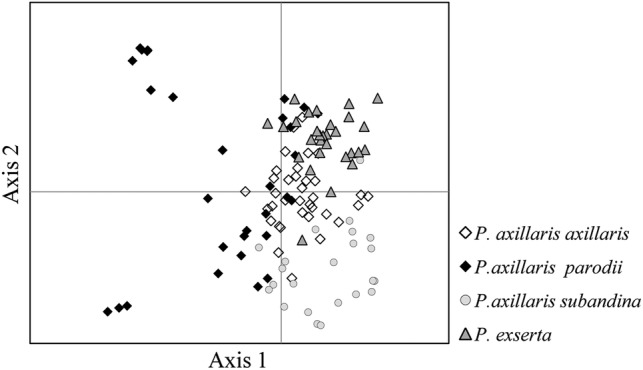
Principal coordinate analysis (PCoA) carried out with the genotypes of the 126 analysed *Petunia axillaris* subspecies and *P. exserta* individuals with 14 EST-SSR markers.

**Figure 3.**
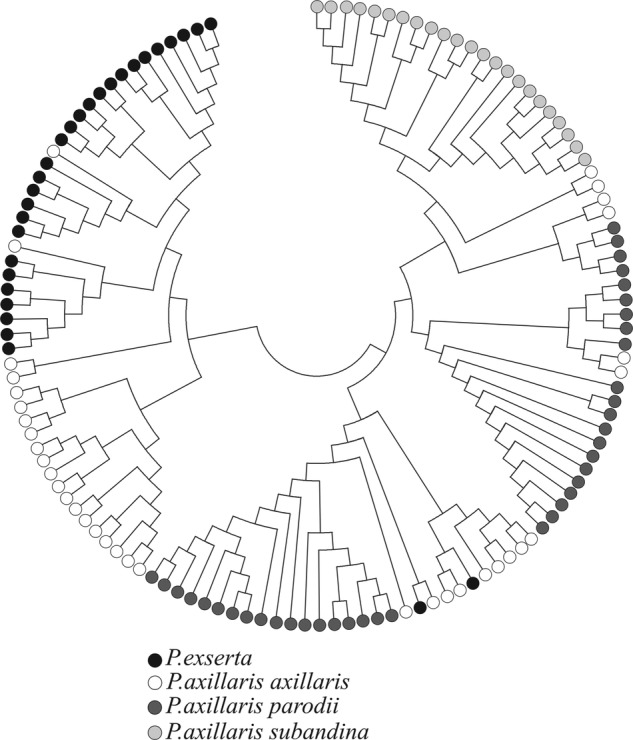
Neighbour-joining tree of 126 individuals (coloured by taxon), constructed based on a matrix of microsatellite alleles shared among individuals.

Principal coordinates analysis (Fig. 2) was conducted to assess the clustering of individuals based on EST-SSR polymorphisms. Plotting the first two axes showed that *P. axillaris* ssp*. axillaris* and *P. exserta* were closely related in the coordinate space and that *P. axillaris* ssp*. parodii* formed a wider scattered group, whereas the other two subspecies each formed only one exclusive group. The NJ tree (Fig. 3) of the 126 individuals obtained from a matrix of shared microsatellite alleles presented two major clusters: the first grouped *P. axillaris* ssp*. axillaris* and *P. exserta* individuals (Group A); whereas individuals of *P. axillaris* ssp*. parodii* and *P. axillaris* ssp*. subandina* preferentially composed the second group (Group B). Group A was divided into two subgroups, one corresponding to *P. exserta* individuals and only two individuals of *P. axillaris* ssp*. axillaris* and another composed of only individuals of the *P. axillaris* ssp. *axillaris*. Some *P. axillaris* ssp*. axillaris* and *P. exserta* individuals clustered with Group B (Fig. 3). The tree topology showed a close relationship between *P. exserta* and *P. axillaris* ssp*. axillaris*, in agreement with PCoA results.

### Evaluating hybrid individuals

**Figure 4.**
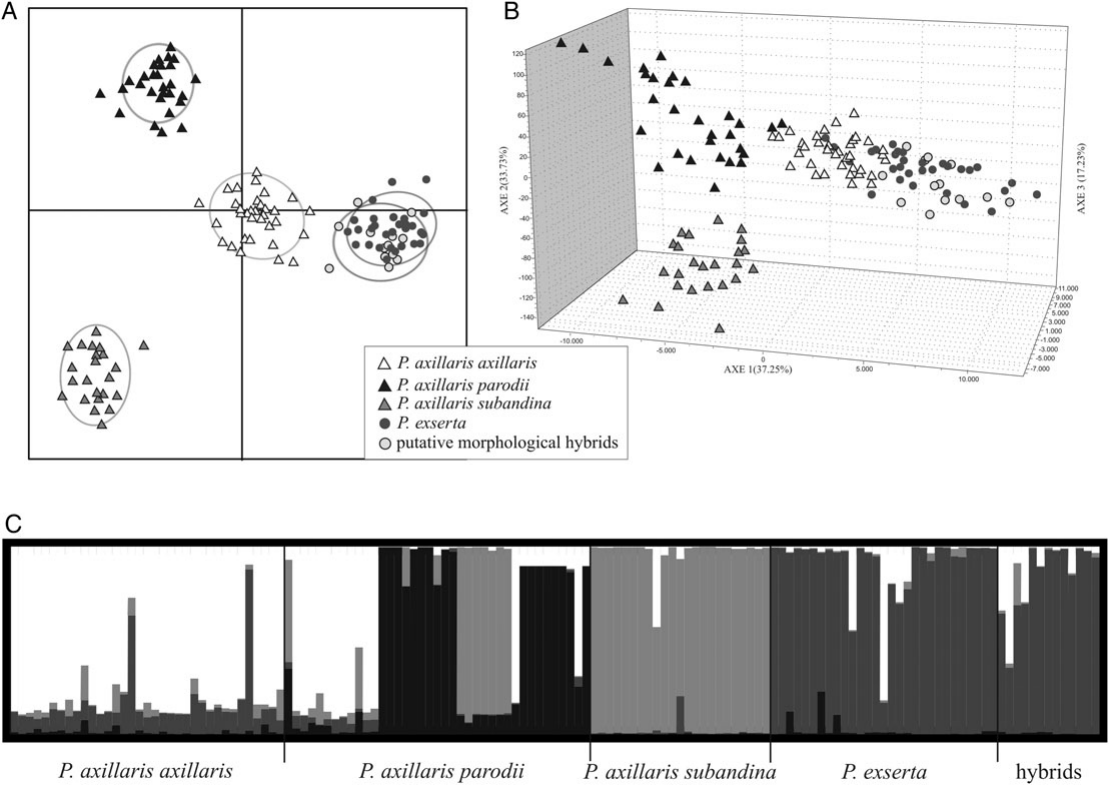
Genetic characterization of putative hybrids. (A) Discriminant analysis of principal components (DAPC) showing the two principal components. Different taxa are indicated according to the legend (A and B). (B) Diagram of the FCA presenting the individuals in a multidimensional space. (C) The estimated proportion of membership in the corresponding clusters (*K* = 4), as calculated using the STRUCTURE software.

The polymorphism of these 14 EST-SSR loci indicated that putative hybrid individuals with a morphology between *P. axillaris* ssp. *axillaris* and *P. exserta* presented similar genetic profiles to those observed for morphologically typical *P. exserta* or *P. axillaris* ssp*. axillaris* individuals (Fig. 4). In the DAPC (Fig. 4A) morphological hybrids grouped more closely to *P. exserta* individuals. Typical *P. exserta* individuals were more close related to *P. axillaris* ssp*. axillaris* than to the other *P. axillaris* subspecies. In the FCA (Fig. 4B) the proximity of *P. axillaris* ssp*. axillaris* and *P. exserta* was more apparent, and the hybrids were placed within the distribution of these taxa. This analysis considers the entire allele to be a representation of one individual for plotting in the hyperspace. The STRUCTURE analysis (Fig. 4C) confirmed these results. In STRUCTURE analysis, the best *K* = 4 was obtained by Evanno's method with the inclusion of putative hybrids in the *P. exserta* group, and the other clusters were associated with the *P. axillaris* subspecies.

## Discussion

In general, EST-SSRs have been found to be significantly more transferable across taxonomic boundaries compared with the traditional ‘anonymous’ SSRs (Tiffin and Hahn 2002; Varshney *et al*. 2005; Woodhead *et al*. 2005). In the present study, we aimed to evaluate a set of microsatellite markers developed from EST-SSRs to obtain information about their cross-transferability in different wild *Petunia* species. We intended to identify markers that can be used in population dynamic analyses, breeding systems and interspecific hybridization events.

Here, we successfully cross-amplified 14 EST-SSR loci and estimated the genetic differentiation of close related wild *Petunia* taxa (the *P. axillaris* subspecies and *P. exserta*). These loci were obtained from an EST database with sequences that were isolated preferentially from the roots and floral organs of *Petunia hybrida* (Bossolini *et al*. 2011).

Overall, *P. axillaris* ssp*. axillaris* had a higher number of alleles per locus and allelic richness (Tables 2 and 3), which illustrated the high diversity of this taxon. This is consistent with the findings of previous studies of plastid sequences, cleaved amplified polymorphic sequence (CAPS) nuclear markers (Segatto *et al*. 2014*a*; Turchetto *et al*. 2014*a*), and the phylogenetic position of these taxa (Reck-Kortmann *et al*. 2014). The mean number of alleles per locus obtained using EST-SSRs in these species (10 for a complete data set) was higher than that obtained in *Petunia integrifolia* based on genomic SSRs (a mean of three alleles per locus; Kriedt *et al*. 2011). A recent study involving wild *Petunia* species and commercial *P. hybrida* (Segatto *et al*. 2014*b*) showed that *P. integrifolia* species complex share several alleles and also have a low mean number of alleles per locus (ﬁve in *Petunia inﬂata*, four in *P. integrifolia* and three in *Petunia interior*). Here, we found that all taxa contained private alleles, which indicated that EST-SSR loci are a useful tool for evolutionary analyses of these species. The morphological and ecological differentiation between *P. axillaris* and *P. exserta* is clear, and although they share plastid haplotypes, there is no doubt about the placement of these lineages at the species level (Segatto *et al*. 2014*a*). We included samples from different locations to represent the entire geographic distribution of all taxa and to minimize the population effect (population inferences were not included here).

Flower morphology affects the behaviour of pollinators by advertising for reward and restricting access. The maintenance of morphological traits is responsible for preserving the genetic boundaries between species, although past hybridization events could have occurred. The individuals mentioned in this study presenting an intermediate morphology were considered to be interspecific hybrids due to their morphological traits—especially the corolla colour and position of reproductive organs, plastid polymorphism and positional distribution inside caves. These individuals had been analysed previously and were considered hybrids between *P. exserta* and *P. axillaris* (Lorenz-Lemke *et al*. 2006). The putative hybrids presented alleles that were private to *P. axillaris* ssp*. axillaris* or *P. exserta* (Table 3), thereby highlighting the ability of these markers to identify hybridization events between these species. However, only one marker private to *P. axillaris* ssp. *axillaris* and *P. exserta* was shared between hybrid individuals (PM188; **Supporting Information—Table S2**). These results provide evidence that the putative hybrids evaluated may not be F1 hybrids and could have backcrossed with the parental species or crossed among themselves. Studies analysing the morphology of individuals, in addition to canonical morphological data coupled with analysis of these markers, can be useful for understanding the evolution of these closely related wild species. In the STRUCTURE analysis (Fig. 4), putative hybrids showed signs of admixture with *P. axillaris* ssp*. axillaris*, but the same level of admixture has been found in the *P. exserta* group. This pattern could be explained by backcrossing with parental species or by shared ancestral polymorphisms. Moreover, the putative morphological hybrids occurred only inside caves (*P. exserta* habitat) and it is possible that the hybrids have habitat or ecological restrictions. Another more controversial possibility is that speciation with gene flow occurred, and some of the morphological traits were not uniform as demonstrated to other species (Wu 2001). The consequences of hybridization differ depending on whether the species diverged with gene flow or underwent a secondary contact (Abbott *et al*. 2013). These EST-SSRs enable the unravelling of these evolutionary processes in future population studies.

In *P. axillaris*, the maintenance of morphological traits is responsible for preserving the boundaries of the subspecies. The clear ecological and morphological differentiation of the *P. axillaris* subspecies is primarily associated with corolla tube length (Turchetto *et al*. 2014*a*, *b*), and differences in tube length raise interesting questions about the feeding strategy of pollinators. Under controlled greenhouse conditions, *Manduca sexta* moths prefer *P. axillaris* flowers with a larger limb size, which may simply reﬂect its better visibility under low light conditions (Venail *et al*. 2010). Moreover, tube length is associated with a CAPS marker derived from the *Flavonoid hydroxylase* 1 (*HF1*) gene, and the differences between *P. axillaris* subspecies are associated with different genotypes at this locus (Turchetto *et al*. 2014*b*). The *HF1* gene is also informative for distinguishing *Petunia* species (Chen *et al*. 2007). Further experiments, particularly considering morphological traits under ﬁeld conditions, are necessary to evaluate the remaining questions involving *P. axillaris* and *P. exserta* and their pollinators; highly polymorphic EST-SSRs will be informative in these studies.

The close relationship between *P. axillaris* ssp*. axillaris* and *P. exserta* (Kulcheski *et al*. 2006; Lorenz-Lemke *et al*. 2006; Reck-Kortmann *et al*. 2014; Segatto *et al*. 2014*a*) was confirmed and supported by this study. Moreover, the EST-SSRs used were able to differentiate taxa. Our results suggest that these EST-SSR markers may constitute a useful tool for evolutionary and ecological studies involving these species. The original description of these EST-SSRs (Bossolini *et al*. 2011) used very few *Petunia* individuals that had been maintained in a greenhouse for decades. Here, we demonstrated that these markers may cross-amplify wild individuals and are able to differentiate species through private alleles in each species or even by combined genotypes.

Evolutionary studies require many universal primers that can be used in multiple species and that allow comparisons between close related taxa to address questions of population divergence and speciation processes (Noor and Feder 2006). Expressed sequence tag-simple sequence repeats are easily transferable across species (Liewlaksaneeyanawin *et al*. 2003; Chapman *et al.* 2009) and may be a useful tool in evolutionary studies, especially of genera such as *Petunia*, with low genetic diversity and great morphological variability (Fregonezi *et al*. 2013). The EST-SSR markers analysed in *Picea abies* (Fluch *et al*. 2011) presented a high degree of variability and were well suited for an analysis of the stress-related functional variation present in this species. Although EST-SSRs are related to transcribed regions, they can evolve neutrally. In fact, several studies have found that the population structure measures of EST-SSRs are very similar to those derived from anonymous SSRs (e.g. Woodhead *et al*. 2005). Moreover, this type of molecular marker has been used for hybrid identification, population genetics studies, distinguishing close related taxa and studying functional genetic diversity (Naresh *et al*. 2009; Chapman *et al*. 2010; Barati and Arzani 2012; Li *et al*. 2013; Yang *et al*. 2013).

Expressed sequence tag-simple sequence repeat polymorphisms are associated with transcribed regions of the genome and reflect the genetic diversity inside or adjacent to genes (Clark *et al*. 2003). The major criticism regarding the use of these markers to estimate population parameters is that divergent selection would increase the differentiation among and reduce variability within populations, whereas balancing selection could produce the opposite effect. Chagné *et al*. (2004) showed that estimates of population differentiation based on EST-SSRs are comparable with those based on anonymous SSRs or large-scale comparative analyses. Moreover, only a very small percentage of genes experiencing positive selection has been identified (Li *et al*. 2013; Yang *et al*. 2013). Thus, one should expect that only a small fraction of EST-SSRs would be subject to selection.

In this study, only four loci were found to be under selective pressure. Two of them are of unknown function (PM177 and PM88), while PM192 and PM74 are located in transcription factors **[see Supporting Information—Table S1]**. More analyses, including population studies of each taxon, may clarify the role of these markers in functional differentiation.

The effects of morphological differences on the feeding strategy of the pollinators and adaptive genetic differentiation to maintain the processes of speciation involving close related species with the ability to hybridize in nature raise interesting questions, and *Petunia* species are an excellent model for this area of study. Further experiments, especially analyses of morphology, gene flow and crossbreeding systems, are necessary to answer the remaining evolutionary questions about the *Petunia* species and speciation processes within this genus. Understanding the processes involved in the diversification and speciation of wild *Petunia* species is not only important for ecological and environmental issues but may also be useful for breeding commercial hybrid species; the EST-SSRs tested here constitute useful tools for these purposes.

## Conclusions

The EST-SSR markers scattered throughout the *Petunia* genome are very efficient tools for characterizing the genetic diversity in wild taxa of this genus and are able to classify interspecific hybrids based on the presence of private alleles. These factures indicate that the markers presented here are helpful tools for evolutionary studies.

## Sources of Funding

This work was supported by the Conselho Nacional de Desenvolvimento Científico e Tecnológico (CNPq), the Coordenação de Aperfeiçoamento de Pessoal de Nível Superior (CAPES) and the Programa de Pós-Graduação em Genética e Biologia Molecular da Universidade Federal do Rio Grande do Sul (PPGBM-UFRGS).

## Contribution by the Authors

C.T. and L.B.F. planned, designed and led the project; C.T., J.B. and A.L.A.S. conducted the experiments; C.T., A.L.A.S. and S.L.B. ran the analyses; C.T., A.L.A.S. and L.B.F. wrote most of the text; L.B.F. and S.L.B. provided reagents and equipment to develop the experiments. All authors contributed in the preparation of the study and have commented on and approved the final manuscript.

## Conflict of Interest Statement

None declared.

## Supporting Information

The following additional information is available in the online version of this article —

**Table S1.** Characteristics of 14 EST-SSR markers observed in wild *Petunia* individuals. Forward and reverse primer sequences, repeat motifs, annealing temperatures (Ta), EST GenBank accession numbers, allele numbers (A) for each marker and putative function described in literature are presented for each marker.

**Table S2.** Frequency of private alleles found in each taxa and in putative hybrids of *P. axillaris* ssp. *axillaris* and *P. exserta*.

Additional Information
