# Supplementary material for: Genetic differentiation and hybrid identification using microsatellite markers in closely related wild species
Source: AoB Plants. 2015 Jul 17;7:plv084. doi: 10.1093/aobpla/plv084 (PMC4565426; doi:10.1093/aobpla/plv084)
Supplement: Additional Information [file supp_7_plv084_index.html]

Genetic differentiation and hybrid identification using microsatellite markers in closely related wild species — Additional Information 

# Genetic differentiation and hybrid identification using microsatellite markers in closely related wild species

## Additional Information

Additional Information

- Supplementary Table 1 - docx file
- Supplementary Table 2 - docx file
