# Supplementary material for: Genetic differentiation and hybrid identification using microsatellite markers in closely related wild species
Source: AoB Plants. 2015 Jul 17;7:plv084. doi: 10.1093/aobpla/plv084 (PMC4565426; doi:10.1093/aobpla/plv084)
Supplement: Additional Information [file supp_plv084_plv084supp_table1.docx]

| **Chromossome** | **Locus** | **Primers sequence** | **T_a_ (°C)** | **EST Genebank** | **Repeat**  **motif** | **A** | **Putative**  **Function** |
| --- | --- | --- | --- | --- | --- | --- | --- |
| I | PM101 | F: GAGAGAGAACCCTAACCC | 51 | FN001660 | (CTT)6 | 8 | Unknown |
|  |  | R: GCAGAAGAAACAGAGATCC |  |  |  |  |  |
| I | PM188 | F: CCCAACCATTGGCTACAGCC | 51 | FN037917 | (CTT)8 | 11 | Singlet oxygen response |
|  |  | R: GGACAACACAATACAATCTCTGC |  |  |  |  |  |
| I | PM195 | F: GCCTTTCGCCGCTGTCACTG | 50 | FN026706 | (GAA)6 | 7 | Phosphatidylserine synthase |
|  |  | R: GAGCAAATCGTGACCGTTGG |  |  |  |  |  |
| II | PM21 | F: CTACCGGTAGGCAGTAGTTGC | 50.5 | CV297594 | (TAC)8 | 5 | KDR transcription regulator |
|  |  | R: CCTCGACCTTCTTCCTGAC |  |  |  |  |  |
| II | PM88 | F: CTGTTTCCTAATTACCTG | 51.5 | CV298718 | (TA)7 | 8 | Unknown |
|  |  | R: GCCACTGGCATGGCTGCA |  |  |  |  |  |
| III | PM183 | F: CCTATTTCAGTCCATGAGGC | 51.5 | FN041860 | (GT)10 | 19 | Phosphatase |
|  |  | R: GTTAGCTGTCTGCTGATCAC |  |  |  |  |  |
| III | PM191 | F: GGAGAAGATTGTTGGTAAC | 51 | FN034502 | (TTG)6 | 5 | Transcription factor, GRAS family |
|  |  | R: GGGAAACGATCTCTTGCTG |  |  |  |  |  |
| IV | PM8 | F: TCTGCAAACTTCAAAGCCAA | 50 | CV298210 | (AAGA)10 | 8 | Transcription factor |
|  |  | R: ACATGCCATGCACTTTTGAG |  |  |  |  |  |
| IV | PM173 | F: CAGCGCTATCAACAGCAG | 51 | FN039910 | (GCA)6 | 14 | Clathrin assembly protein |
|  |  | R: GTGAGAGGCAAGTGATTGG |  |  |  |  |  |
| IV | PM74 | F: CACAGGCGACAATACCTCAT | 51.2 | CV299191 | (GA)8 | 5 | Transcription factor |
|  |  | R: GTTCCATCCATTTGGGAAGA |  |  |  |  |  |
| V | PM167 | F: CTCACTAACCAACTTCACC | 52 | FN015039 | (TTC)12 | 12 | Glycosyl transferase |
|  |  | R: CTAAGAAGCTTAAGAGTG |  |  |  |  |  |
| V | PM177 | F: CCCTTACTCTCTTCTTCACC | 50 | FN016759 | (CA)11 | 26 | Unknown |
|  |  | R: GAACTATGAACCATAGCTCTC |  |  |  |  |  |
| V | PM192 | F: GCTGCTTTAAGATTCAGAGGC | 50 | FN036401 | (CAG)8 | 10 | Transcription factor |
|  |  | R: CTGAACTTTGCATTGGC |  |  |  |  |  |
| VII | PM184 | F: GGACTTTTATCAACTACC | 51 | FN027453 | (GAA)6 | 5 | Unknown |
|  |  | R: GCCTTGCCTTTATCGGAC |  |  |  |  |  |
| Total | 14 |  |  |  |  | 143 |  |

**Table S1**. Characteristics of 14 EST-SSR markers observed in wild *Petunia* individuals. Chromosome location; code according Bossolini *et al.* (2011); forward and reverse primer sequences; annealing temperature (Ta); EST GenBank accession number; repeat motif; allele number (A) obtained for all analyzed individuals; and putative function previously published.
