# Supplementary material for: Genetic differentiation and hybrid identification using microsatellite markers in closely related wild species
Source: AoB Plants. 2015 Jul 17;7:plv084. doi: 10.1093/aobpla/plv084 (PMC4565426; doi:10.1093/aobpla/plv084)
Supplement: Additional Information [file supp_plv084_plv084supp_table2.docx]

| **Table S2.** Frequency of private alleles found in each taxon and putative hybrids. | | | | | | |
| --- | --- | --- | --- | --- | --- | --- |
| **SSR marker** | **Allele Size range** | ***P. axillaris* ssp. *axillaris*** | ***P. axillaris* ssp. *parodii*** | ***P. axillaris* ssp. *subandina*** | ***P. exserta*** | **Putative hybrids** |
| PM173 | 157 | 0.015 | - | - | - | - |
|  | 175 | 0.103 | - | - | - | 0.077 |
|  | 178 | 0.015 | - | - | - | - |
|  | 181 | - | - | 0.136 | - | - |
|  | 187 | 0.015 | - | - | - | - |
| PM177 | 202 | - | - | 0.205 | - | - |
|  | 208 | - | - | 0.109 | - | - |
|  | 212 | - | - | 0.043 | - | - |
|  | 216 | - | 0.013 | - | - | - |
|  | 220 | - | - | 0.065 | - | 0.083 |
|  | 222 | - | - | 0.043 | - | - |
|  | 250 | - | - | - | 0.125 | - |
|  | 252 | - | - | - | 0.125 | - |
|  | 254 | - | - | - | 0.018 | 0.042 |
| PM188 | 115 | 0.059 | - | - | - | - |
|  | 121 | - | - | - | 0.071 | 0.077 |
|  | 142 | 0.118 | - | - | - | 0.115 |
|  | 145 | 0.015 | - | - | - | 0.038 |
|  | 151 | - | 0.026 | - | - | - |
| PM183 | 126 | - | 0.051 | - | - | - |
|  | 136 | 0.029 | - | - | - | - |
|  | 139 | 0.015 | - | - | - | - |
|  | 148 | - | 0.013 | - | - | - |
|  | 164 | 0.029 | - | - | - | - |
|  | 169 | - | - | - | 0.017 | - |
|  | 172 | - | - | - | 0.017 | - |
|  | 174 | 0.059 | - | - | - | 0.038 |
|  | 178 | - | 0.013 | - | - | - |
| PM184 | 90 | - | - | 0.043 | - | - |
| PM191 | 173 | 0.043 | - | - | - | - |
| PM88 | 134 | - | - | - | - | 0.038* |
|  | 164 | - | - | - | 0.167 | 0.462 |
|  | 168 | - | 0.077 | - | - | - |
|  | 174 | 0.014 | - | - | - | - |
| PM21 | 134 | 0.057 | - | - | - | 0.038 |
|  | 137 | 0.029 | - | - | - | - |
|  | 140 | - | - | - | - | 0.038* |
|  | 143 | - | - | - | - | 0.077* |
| PM8 | 163 | 0.129 | - | - | - | 0.154 |
|  | 167 | 0.057 | - | - | - | 0.077 |
|  | 191 | 0.014 | - | - | - | - |
| PM74 | 200 | - | 0.026 | - | - | - |
| PM195 | 208 | 0.043 | - | - | - | - |
|  | 229 | - | 0.029 | - | - | - |
| PM192 | 224 | - | 0.031 | - | - | - |
|  | 260 | - | - | - | 0.017 | - |
| PM167 | 279 | - | - | - | 0.034 | 0.038 |
| PM101 | 240 | 0.014 | - | - | - | - |
|  | 258 | 0.014 | - | - | - | - |
|  | 276 | 0.014 | - | - | - | - |
| **Total** |  | **22** | **9** | **7** | **9** | **12 (3)** |
| Note: In hybrid individuals we found 12 private alleles shared with one or other taxa, and 3 private alleles found only in  hybrid individuals (*). | | | | | | |
